# Medical Education in Texas

**Published:** 1897-06

**Authors:** James Orr

**Affiliations:** Terrell, Texas


For the Texas Medical Journal.

### MEDICAL EDUCATION IN TEXAS.

BY JAMES ORR, M. D., TERRELL, TEXAS.

[Read before the Terrell Medical Society.]

STATE Legislatures are composed of fallible beings like ourselves, hence cannot be expected to reach perfection in all their enactments; however, it does seem that glaring incongruities should be seen and intelligently remedied, when the effect of such legislation is a matter of daily comment and general knowledge. To none of our statutes do these remarks apply with more force than to those regulating the practice of medicine, and medical education.

I presume, Mr. President, that every member of this society is familiar with the law regulating the practice of medicine in Texas, and with the fact that the certificate of the District Medical Examining Board is the highest authority in the State. Also that the State has a medical school of its own, besides chartering several other medical educational institutions. Now let us see the practical workings of these laws.

The State, following up the paternalistic idea, so rampant just now, has established a medical school at Galveston, which, with its costly building and large corps of high-priced teachers, invites the youths of Texas to leave the plow, the workshop and the counter for the purpose of qualifying for one of the so-called learned professions, practically without money and without price.

It would seem that one medical school in Texas would be sufficient, but there is so much rivalry among our pushing cities that no one of them seems willing to allow the other to have any exclusive attraction, hence the citizens of Fort Worth were grievously unhappy until about all the doctors there got together and established a Medical University! To meet with any success at all the fees of this institution were placed so low that each *professor* lectures for about the same price charged for visits to private patients. But it had to be done; the State's prices had to be met, besides competition from rival towns must be prevented. Fort Worth must be the chief, if not sole, rival of the State in the cheap medical education business.

Not content with the facilities offered by our cheap universities, the State has authorized every district judge to appoint a medical examining board, whose certificate is nothing more nor less than a license to practice medicine in Texas, the State's seal of approval upon the qualifications of the holder to deal with the intricate problems of disease, to be intrusted with the administration of dangerous drugs, to hold human life, health and happiness in his hands. We have more than fifty of these medical diploma mills grinding out certificates to the learned and unlearned at prices far below the cheap rates of Galveston and Fort Worth, with those medical institutions they see in constant rivalry, and whose rules, regulations and requirements they unhesitatingly set at naught. To illustrate this, I suppose you have all read of the episode of Fort Worth a few weeks ago, when a large number of the class in the medical school there, not having fulfilled the requirements for graduation, and, in the judgment of the faculty, with whom they were in daily contact, not sufficiently learned to practice medicine, went before one of the district boards in a body and were duly licensed as qualified physicians, thus obviating the necessity for further medical education, and saving the time and low fees required by the school they were pretending to patronize. Is it possible for any serious matter to be more farcial than this?

The climax of this cheap medical education business was not reached, however, until the State chartered a medical college at Gatesville, where a man and his wife, without hospital, dissecting room, library, college, lecture room or *class*, issue diplomas to *all* who *send* them the required fee in advance.

Thus Texas *regulates* the practice of medicine, and it is no excuse to preach about the survival of the fittest, about the people recognizing and rewarding merit, or the democratic right to patronize whom ever they wish, whether qualified to practice or not; the fact remains the same. This is an era of paternalism, when the people expect the State to do everything for them, including certifying to the qualifications of those who propose to deal with life and death, hence it owes it to them to place its seal of approval upon those only who by study and observation have fitted themselves for a work so important and so full of responsibilities. When the State does this a higher and more perfect system of medical education will exist, only those having special tastes in that direction will enter the profession, and medicine will be elevated from the debased position of a cheap trade to that of a learned basis. Then will disease be intelligently studied and combatted, correct ideas of health and disease be inculcated, and the people educated upon questions wherein quacks, charlatans and demagogues now prosper through their ignorance.

If the State of Texas desires to legislate in the interests of the common people let it do away with the present statutes on the subject and hereafter license those who, by long study and training, are qualified to practice medieine. Let the requirements be uniform throughout the State, then will the people be educated and protected.

